# The Immune Cells in the Development of Oral Squamous Cell Carcinoma

**DOI:** 10.3390/cancers15153779

**Published:** 2023-07-26

**Authors:** Vito Carlo Alberto Caponio, Khrystyna Zhurakivska, Lorenzo Lo Muzio, Giuseppe Troiano, Nicola Cirillo

**Affiliations:** 1 Department of Clinical and Experimental Medicine, University of Foggia, 71122 Foggia, Italy; vitocarlo.caponio@unifg.it (V.C.A.C.); khrystyna.zhurakivska@unifg.it (K.Z.); lorenzo.lomuzio@unifg.it (L.L.M.); giuseppe.troiano@unifg.it (G.T.); 2 Melbourne Dental School, The University of Melbourne, Melbourne, VIC 3010, Australia; 3 School of Dentistry, University of Jordan, Amman 11942, Jordan

**Keywords:** lymphocytes, macrophages, oral cancer, oral potentially malignant disorders, tumor microenvironment, tumor immunoediting

## Abstract

**Simple Summary:**

The cells of the immune system can exert a dual effect on cancer development and growth. On the one hand, the immune system can be activated by tumor antigens and can elicit an antitumor response. On the other, the inflammatory milieu in the tumor microenvironment can trigger immune effector mechanisms that promote tumor growth. In the oral cavity, the balance between protumor and antitumor immunity can influence the progression from premalignancy to carcinoma. In this article, we review the cells and mechanisms that are thought to be the most important immune determinants of oral cancer development and progression.

**Abstract:**

A still unresolved issue surrounding tumor formation concerns the role that the immune system plays in preventing the formation and progression of neoplasia, including oral squamous cell carcinoma (OSCC). Antitumor immunity has historically been seen as a critical barrier for cancer cells to develop, grow and spread, and this can be modulated using immunotherapies to achieve antitumor clinical responses. However, it has recently become clear that tumor-associated immunity, particularly the inflammatory microenvironment, has the paradoxical effect of enhancing tumorigenesis and progression. In this review, we discuss the multifaceted function of infiltrating immune cells in suppressing or promoting premalignancy and cancer. In particular, we report on the evidence supporting a role for T lymphocytes, dendritic cells, macrophages, and neutrophils in the development and progression of oral potentially malignant disorders (OPMD) and OSCC. We also draw attention to the clinical relevance of immune cell phenotypes and associated molecules for use as biomarkers and to the translatability of current research findings to improve classification systems and precision medicine in patients with OSCC.

## 1. Introduction

Head and neck cancer (HNC) is one of the most common malignancies worldwide, with the majority of HNCs arising from the stratified epithelium of the oral cavity. Worryingly, over 300,000 new cases of oral squamous cell carcinoma (OSCC) are diagnosed every year, particularly in the Indian subcontinent and Southeast Asia, where OSCC is overall the third most common type of cancer [1]. Current evidence supports the existence of a multi-step process of oral carcinogenesis, which clinically translates into the onset of oral potentially malignant diseases (OPMDs) in the early stages of progression to malignancy. OPMDs eventually progress to oral cancer following the acquisition of a number of additional mutations [2]. Thanks to the relatively easy inspection of the oral cavity, it is possible to detect early changes in the oral mucosa before these progress to overt malignancy, and thus reduce mortality [3]. However, while oral carcinogenesis consists in an accumulation of genetic and epigenetic alternations, growing evidence calls into question the role of the surrounding microenvironment in the development of malignancy [4].

**Figure 1:**
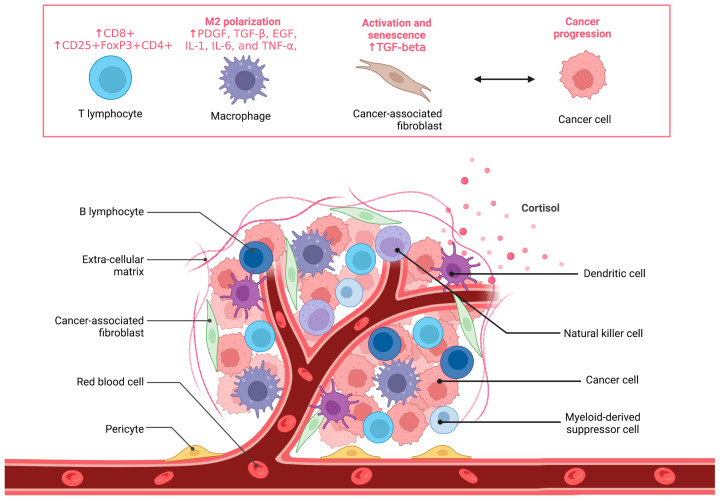
Graphical representation of cancer tissue. In addition to malignant cells, cancer tissue includes extra-cellular matrix components, blood vessels and non-malignant cells. The cell types depicted on top of the panel (T lymphocytes, macrophages and activated fibroblasts) play a key role in cancer progression.

The microenvironment is a complex milieu of extracellular matrix components, blood vessels and non-malignant cells, such as immune cells and fibroblasts, that surround the tumor [5] or its pre-malignant lesion (Figure 1). The continuous exchange of different signals among these components leads to changes in the environment, which keep evolving during the progression from normal to cancerous tissue [6]. For example, we have shown that stromal fibroblasts found in cancer tissues (cancer-associated fibroblasts, CAFs) promote epithelial cancer progression via paracrine mechanisms that involve oxidative stress and cellular senescence [7,8].

As knowledge of cancer mechanisms progresses, it has become apparent that the normal cell progressively evolves to a neoplastic state by evading growth suppression, enabling replicative immortality and resisting apoptosis (cell-intrinsic mechanisms) as well as via cell-extrinsic mechanisms, such as escape of immune surveillance [9]. In this scenario, inflammation has acquired a paradoxical role, since the immune cells involved in the elimination of altered cells may be educated to promote carcinogenesis by different mechanisms [10]. In agreement with this view, higher incidence of OSCC is observed in OPMDs characterized by chronic inflammation [11], and concomitant production of anti-inflammatory, immunosuppressive molecules is observed in cancer tissues, including OSCC [12]. Strikingly, cancer cells can themselves produce immunomodulatory molecules, such as cortisol [13], hence driving the immune response to facilitate or repress cancer growth and immune surveillance.

In this review, we will examine the immune cells involved in OSCC-associated inflammation, their role in oral carcinogenesis, as well as their clinical significance from a prognostic standpoint.

## 2. Immune Cells of the Tumor Microenvironment in Oral Cancer Development and Progression

The idea that the immune system can recognize and destroy nascent-transformed cells finds its roots in the nineteenth century with Virchow’s work and was later conceptualized in the cancer immunosurveillance hypothesis of Burnet and Thomas [14]. In the last 30 years, this hypothesis has been substantiated by a growing body of experimental evidence. Interestingly, this work has shown that the immune system can also function to promote or select tumor variants with reduced immunogenicity, thereby providing cancer cells with a mechanism to escape immunologic detection and elimination. These findings have led to the development of the cancer immunoediting hypothesis, which acknowledges both the antitumor and tumor-promoting actions of the immune system in tumor development. Here, we discuss the role that immune cells play in the multistep process of oral carcinogenesis.

### 2.1. T Lymphocytes

A key immune-modulatory element in oral precursor lesions is the tumor-infiltrating lymphocytes (TILs), particularly CD8+ T lymphocytes, which are abundant in OPMDs [15]. Several studies have revealed an association between TILs and higher grading in dysplastic lesions [16,17,18]. In one example, the ratio between CD8+ T lymphocytes and CD4+ cells increased following dysplastic change, which may represent an attempt of the immune system to eliminate altered cells [18,19]. Similarly, Gannot et al. found an increase of CD4+ CD8+ T lymphocytes and B cells in moderate and severe dysplasia and OSCC compared to hyperkeratotic lesions [20], suggesting that antitumor immune response mounts during the development of OSCC. Conversely, IgA and IgG-secreting B cells were found in leukoplakia with dysplasia and were decreased during the progression to malignancy [21], which might signal a reduction in humoral antitumor immune surveillance.

Crucially, Strauss et al. demonstrated a switch towards an infiltrating CD25+FoxP3+ CD4+ phenotype in patients with HNSCC. This unique subpopulation of T cells secreting interleukin-10 (IL-10) and transforming growth factor (TGF)-β1 mediates immune system suppression in the tumor microenvironment and hence favours pro-tumour immunity [22]. Consistently, other studies have found that CD25+ and FoxP3+ lymphocytes were associated to OPMD progressing to OSCC [15,23,24]. Gan et al. recently performed an immunohistochemical and transcriptomic profiling of diverse severity OPMDs and early OSCCs. They found that infiltrating lymphocytes were present in 80% of high-risk OPMDs and OSCCs, compared to 9% of benign lesions. In high-risk OPMDs, transcriptomic profiling revealed the existence of T-cell inflamed and non-immune reactive subtypes. T-cell inflamed subtype was characterized by T lymphocytes, interferon and PD/PD-L1 pathway signatures, suggesting the presence of an impaired immune surveillance [25]. This study supported the results from Yagyuu et al., who found increased immunohistochemical expression of PD-L1, CD163+ macrophages and CD8+ lymphocytes in high-grade dysplasia [12].

The results of these studies have informed the recent development of an immunoreactivity score based on the expression of PD1, PD-L1, FoxP3, IL-6, IL-10 and TGF-β1. A higher number of T-reg cells and the expression of such markers correlated to higher grades of dysplasia in OPMDs and the strongest correlation was found between PD1 and PD-L1. Collectively, these results would suggest that immune suppression and PD1/PD-L1 axis are instrumental in the progression of OPMDs to OSCC [17].

### 2.2. Macrophages

Tumor-associated macrophages (TAMs) are the most abundant immune cells in the tumor microenvironment and act in concert with TILs. In particular, complex interactions between lymphocytes and macrophages can either promote or contrast the formation of a pro-inflammatory and immunosuppressive environment [26].

Oral dysplastic lesions can recruit macrophages by expression of human beta-defensin 3 [27], and an increase in macrophages and myeloid-derived suppressive cells (MDSCs) correlates with malignant progression, in particular by M2 macrophage polarization [12,24,28,29,30,31]. In several studies, M2 were characterized by CD163+ and CD204+ and correlated with the presence of FoxP3 and CD25+ lymphocytes; specifically, both cell types increased with the worsening of dysplasia [15,30]. Despite the strong evidence of a pro-tumor activity of M2 macrophages [32], conflicting results have also been reported. In contrast to their own results in a smaller cohort of patients, Yagyuu et al. could not confirm a prognostic role of M2 CD163+ in malignant transformation although some differences in study design and inclusion might have led to this inconsistency [12,33]. In a mouse study, M2 signatures were predictive of longer oral cancer-free survival [34]. Nevertheless, canonical markers of M2 might be functionally associated to an M1 phenotype. For example, Weber et al. found CD163+ macrophages co-expressing CD11c, which is also known to be a marker for M1 polarization [29]. Mori et al. observed an increase of CD163+ macrophages in higher grade dysplasia, but this occurred in an immunosuppressive environment regulated by CD4+ lymphocytes and associated with the expression of CXCR3, CCR5, CXCL9, STAT1 and interferon-induced gene products [35].

The role of external etiopathogenetic factors in the modulation of the cancer immune response has also been considered. For example, smoking was shown to determine an immunosuppressive environment characterized by M2 infiltration, arginase-1 and IL-10 and lower TNFα and iNOS [36]. Stasikowska-Kanicka et al. found an increase in CD68+, CD163+ (M2), iNOS+ (M1), CD4+, CCR4+ (Th2) and CCR5+ (Th1) in the progression to metastatic OSCC, whereas the presence of CD8+ cells negatively correlated to both CD163+ and iNOS+ macrophages regardless of the presence of metastasis [37]. Ye et al. showed that signal regulatory protein α (SIRPα) correlated with the number of CD68+ macrophages while advancing from normal to OPMD to OSCC, while CD163+ negatively correlated with SIRPα expression. In an in vitro model of co-culture of macrophages with oral cancer cells, the blockade of SIRPα led to M2 polarization with inhibition of phagocytosis, IL-6 and TNF-α, and secretion of IL-10 and TGF-β [38]. Hence, M1-M2 phenotype switch might be overlapping during the whole process of oral carcinogenesis [39]. Moreover, another important detail to be considered is that all these different studies evaluated and characterized different makers of expression and different tissue localization of macrophages, such as stroma, periphery, sub- and intraepithelial which may contribute to these contrasting results and differential M1-M2 phenotype characterization [12,18,28,29,30,33,35,37,39,40,41].

### 2.3. Dendritic Cells

While macrophages and lymphocytes may be considered the main characters of the immune microenvironment, many other cells types contribute to immunosurveillance, and their reciprocal interaction may promote or suppress OPMD/OSCC development [42]. In the context of mucosal microenvironment, dendritic cells (DCs) are involved in major histocompatibility complex call I pathway by presenting antigens to T cells [43,44]. The most abundant type of DC in the oral cavity is dendritic Langerhans cells (LCs) [45] and current evidence demonstrates contrasting roles in malignant progression. Specifically, several studies found an increase of CD1a+ LCs while progressing from normal to mild and severe dysplasia [18,45,46,47,48,49] while others found a decrease of this cell population [50,51]. Of interest, in the study of Wang et al., nine patients going towards malignant transformation reported significantly lower LCs. It is plausible, therefore, that changes in the number of infiltrating LCs affects the immunosurveillance ability during early carcinogenesis [46,51].

### 2.4. Mast Cells

Another supporting component in this multicellular microenvironment are mast cells (MCs), which have been emerging as possible players in the malignant progression from OPMD to OSCC. Telagi et al. found an increase of MCs in patients with dysplasia and oral submucous fibrosis, which was particularly prominent in the presence of inflammation [52]. Mast cells were also increased in leukoplakia, lichen planus and actinic cheilitis [47,53,54,55,56,57,58]. Mechanisms by which MCs might contribute to malignant progression are still not well understood [52,59]. Piecemeal degranulation is an unconventional secretory pathway characterized by vesicular transport of small packets of selected materials from the cytoplasmic secretory granules to the cell surface and might represent one of the main mechanisms by which MCs contribute to cancer progression. These granules may be enriched of cytokines, proteases, arachidonic acid derivates and growth factors, representing a heterogeneous phenotypic expression of MCs [59,60,61,62]. A systematic review on this topic showed that the highest number of MCs was found in OPMD while their presence declined in OSCC. Indeed, it appears that MCs are abundant in an inflammatory tumor microenvironment although it is not clear whether these cells exert an anti- or pro-tumor activity [59]. Several studies found an increase of MCs’ density while moving from normal mucosa to dysplasia to OSCC, which positively correlated to microvessel density (CD34+/CD31+). This would suggest MC activity parallels neo-angiogenesis [63,64,65,66,67,68,69,70,71]. Oliveira-Neto found a decrease of MCs in OSCC and transforming OPMDs, as a consequence in microenvironment changes, and reported an inhibition of MC migration that could reflect impaired control during tumor initiation [72]. Similar results were documented by Singh et al. [73]. This dichotomous role may reflect an attempt of the immune system to fight altered cells by promoting cytotoxic effect in the early phases; however, once the tumor is established, MCs become educated to produce pro-angiogenic and pro-tumoral factors [59].

### 2.5. Myeloid-Derived Suppressive Cells (MDSCs)

MDSCs have recently gained a more prominent role in the pro-tumorigenic tumor microenvironment, although current studies are limited to patients with a diagnosis of OSCC or in vivo animal models of OPMD [28,74]. MDSCs’ accumulation and malignant progression was associated to porphyromonas gingivalis infection by increase in CXCL2, CCL2, IL-6 and IL-8, which drew malignant progression [75]. The presence of MDSCs also correlated with CD4+FoxP3+ lymphocytes and IL-1β secretion in a dectin-1 depending signal in a model of fungal infection [76].

### 2.6. Neutrophils and Eosinophils

Scarce evidence exists with regards to neutrophils and eosinophils as very few studies have explored their role in the malignant progression of OPMDs [77]. Neutrophils were mostly investigated by their ability to form extracellular traps (NETs) which was observed to be a prominent ability in patients with oral lichen planus, thus suggesting a possible role in the progression to OSCC [78]. Eosinophils were also found increased in patients with leukoplakia and the mean number correlated to the advancement of dysplasia [77] although this finding was not confirmed in other studies [65,79,80].

### 2.7. The Immune Function of Cancer-Associated Fibroblasts

Although not properly considered part of the immune system, fibroblasts represent the main cellular component of extracellular matrix and are known to produce several cytokines with emerging roles in innate immune response [81,82,83]. Fibroblasts/CAFs are prominent cell types in the tumor microenvironment and hence could interfere in the immunosurveillance mechanisms in cancer involving a cross-talk with primary immune cells [84,85,86,87]. While these α-smooth muscle actin positive (α-SMA) (myo)fibroblasts are virtually absent in the normal oral mucosa [88,89], it has been reported that myofibroblasts significantly increase in number while progressing to OPMD and to OSCC [88,89,90,91,92,93]. Other authors could not find evidence of myofibroblasts in normal mucosa or in patients with oral leukoplakia or dysplasia but only in OSCC [94,95,96]. Current evidence shows a wide heterogeneity in the role of myofibroblasts in the progression to malignancy [97]. In this regard, a recent systematic review suggested that myofibroblasts may be involved in the progression of oral submucous fibrosis but not in patients with leuko-erythroplakia [97]. Indeed, it has been reported that increased inflammatory response in the stroma is inversely associated to myofibroblasts [98,99], and OSCC—fibroblasts contact is necessary for induction of myofibroblast phenotype [100,101]. Conversely, findings from our group show that a paracrine cross-talk between malignant keratinocytes and fibroblasts is sufficient to drive tumor migration and invasion in a TGF-β-dependent manner [7,8]. These data are in line with the results obtained in an in vitro model where OSCC cell line was able to transdifferentiate fibroblasts to myofibroblasts via secretion of TGF-β1 [99].

## 3. Immunopathogenic Mechanisms in OSCC and Precursor Lesions

Although a functioning immune system is instrumental for the elimination of neoplastic cells, when the immune response becomes regulated by the tumor microenvironment, this produces an opportunity for the development of malignant cells that are capable of escaping the destructive effects of the immune system. The immune microenvironment contributes to tumorigenesis by impairing normal immune cell activity via immune suppression and tolerance, as well as by enhancing angiogenesis and ROS production [102,103,104].

The mechanisms of this dual pro- and anti-cancer role of the immune cells will be discussed here.

### 3.1. Acquisition of Tolerance during the Progression to Malignancy

While forming cancer cells express antigens that are recognized and targeted by the immune system, it is more difficult to explain why a similar immune dysregulation is witnessed in OPMD. One fascinating hypothesis on how transforming cells evade the immune surveillance is that precursor lesions express tumor antigens, which facilitate the acquisition of tolerance over time. Consistently, a pattern of different tumoral antigens has been shown in OPMDs, such as MAGE cancer testis antigens, NY-ESO-1, MUC1 and neo-antigens [105,106,107]. It is possible that the emergence of CD25+ and FoxP3+ lymphocytes observed in OPMD progressing to OSCC [15,23,24] might represent the immune system adaptation and education provided by the transformation of premalignant cells [28]. One of these adaptive changes includes CD8+ T-lymphocytes’ ability to recognize antigens and deliver their cytotoxic effect [108]. The functional activity exerted by cytotoxic CD8+ T-lymphocytes in this context has not been definitively proven [109]. More likely, inflammation may promote genomic instability in the early stages; while later, once genomic changes have occurred, new mechanisms might exert an immunosuppressive conditioning, which facilitates the progression from OPMD to over carcinoma [28,110].

### 3.2. Expression of Immune Checkpoint Markers

Immune checkpoints are stimulatory and inhibitory pathways that modulate the immune response while maintaining self-tolerance. However, the expression of some of these immune-checkpoint proteins by malignant epithelial cells (rather than immune cells) dysregulates the antitumor immunity and favors the growth and expansion of cancer cells. In the context of oral carcinogenesis, it has been shown that the elevated number of T-reg cells and the expression of immune checkpoint markers correlated to higher grades of dysplasia in OPMDs, with the strongest correlation found between PD1 and PD-L1 [19]. This suggests that the PD1/PD-L1 axis may be responsible for the progression of OPMDs to OSCC, possibly leading to T-cell exhaustion and immunosuppressive environment [17]. Strategies that target these regulatory pathways to enhance immunological activity against tumor cells are being developed, with mixed results [111,112]. The most successful immune checkpoint blockade strategy is anti-PD-1/PD-L1 therapy that has been approved to treat a wide variety of cancer types, such as blood, skin, lung, liver, bladder and kidney cancers. While the more durable response of checkpoint blockade compared to chemo- or targeted therapies may be related to mechanisms of immunological memory, a relatively low response rate has been observed in most cancers, including HNSCC [113]. Therefore, further immune checkpoint inhibitors besides those targeting the PD-L1/PD-1 pathway need to be explored for therapeutic use.

### 3.3. Role of Cancer Stem Cells in Immune Evasion

**Figure 2:**
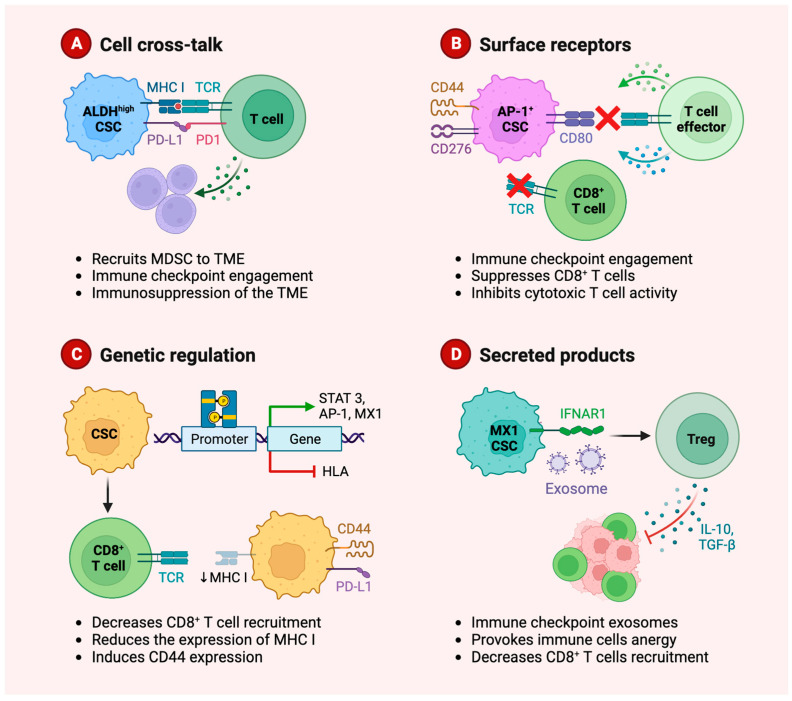
Graphical representation of the mechanisms of immune evasion by cancer stem cells (CSCs). CSCs promote an immunosuppressive microenvironment via direct interaction with T cells (**A**,**B**), genetic regulation leading to reduced immunogenicity (**C**) and paracrine mechanisms provoking immune cells anergy and suppression of the immune response (**D**). MDSC, Myeloid-derived suppressor cells; TME, tumor microenvironment.

Cancer stem cells (CSCs) play a critical role in carcinogenesis, progression to metastasis, and resistance to antineoplastic treatment of head and neck tumors [114]. Since CSCs act as tumor-initiating cells, these cell populations may develop intrinsic mechanisms to evade immune surveillance both via direct contact and paracrine regulation of immune cells involving secreted molecules and exosomes (Figure 2). 

Studies have shown that CSCs express low levels of molecules involved in processing and presenting tumor antigens to T cell receptors (TCRs), a crucial stimulatory signal to T-cell response, and escape from recognition by anti-tumor immunity [115]. In addition, enriched PD-L1 expression in CSCs has been suggested to facilitate CSC immune evasion in head and neck cancers by suppressing T-cell-mediated immunity [116]. Subsequent studies characterized these TIL subsets and found that T cell infiltration was enriched in an effector memory phenotype (CD45RA−/CCR7−). Naïve T cells (CD45RA+/CCR7+) were decreased in the microenvironment compared to PBMC of patients while regulatory T cells (CD4+/CD25+/CD127 low and CD4+/CD39+) were elevated [117]. Notably, the immunomodulatory molecule cortisol induces the formation of stem cell-like populations in epithelial cancers [118] and glucocorticoid receptor inhibition by mifepristone mediates anti-proliferative effect on ovarian mesenchymal stem cells [119]. It is possible, therefore, that cancer-derived cortisol promotes immune evasion both directly by immune suppression and indirectly by enriching CSC subpopulations.

### 3.4. Role of Immune Modulatory Cytokines

The role of cytokines in cancer, including OSCC, has been studied extensively and the main effector molecules regulating the pro-tumor immune activity are CSF-1, IL-6, VEGF, PGE-2, TGF-β and IL-10 [120]. The immune modulatory role of these cytokines in the tumor microenvironments of head and neck squamous cell carcinomas has been reviewed in detail recently [121]. Notably, the clinical prognostic value of tumor-infiltrating cell types depends on their secretory cytokine profile. For example, the negative correlation between patient outcome and level of tumor-associated macrophages (TAM) is reliant upon TAM expression of PDGF, TGF-β, EGF, IL-1, IL-6 and TNF-α, which generates a favorable environment for tumor growth [122]. In one example, TGF-β produces an immunosuppressive and tumor-promoting microenvironment in OSCC tissues by stimulating the production of Treg cells and CAFs, which then results in the inhibition of cytotoxic T lymphocytes (CTLs) and natural killer cells. We have shown that CAFs, in turn, produce high levels of TGF-β1 and TGF-β2 and promote oral carcinogenesis and local invasion via TGF-β-dependent mechanisms [8,123,124]. Therefore, the tumor-limiting vs. tumor-promoting action of cytokines is not necessarily related to their production by the immune infiltrate; rather, it will result from the interplay between different cell types in the context of tumor microenvironment.

## 4. Clinical Significance of Immune Biomarkers in OSCC

While considerable effort has been directed to the study of the tumor-related host response to OSCC, the principles, mechanisms and molecules governing this process have not been translated to clinical practice to date. In one example, the 8th Edition of the American Joint Committee on Cancer (AJCC) has provided some important novelties in prognostic stratification, including the addition of depth of invasion (DOI) and extranodal extension (ENE) for the evaluation of T (Tumor) and N (Nodal) parameters [125]; however, no immune-related features have been included. Furthermore, prognostication is still based on staging at the time of diagnosis, which only represents a “snapshot” of dimensionality and site involvements of the disease. In other words, the current approach still lacks a “global” assessment of tumor aggressiveness, which makes it difficult to inform precision treatments. For example, tumors presenting at the same stage tend to be treated with the same therapeutic means, yet they show very different biological behaviour and clinical responses. This exemplifies the clinical importance of diving deeper into the discovery and validation of new biomarkers that can reliably predict tumor behaviour and prognosis.

Prognostic biomarkers may be defined as specific biological characteristics that can be quantified at baseline and that could help predict clinical outcomes (e.g., death, recurrence, progression) occurring in the future. A preliminary assessment of future disease behavior may be useful in tailoring clinical decision making and in modulating treatment approaches. The study of the tumor-related immune response seems to be one of the most promising sources of information for prognostic stratification, as it provides information that is not captured by the current TNM classification system, such as the ability of the host’s immune system to fight against cancer cells.

Several studies have proven that tumors at the same stage differ in their immune response capability, which has led to the definition of three different cancer-immune phenotypes: the immune-desert phenotype, the immune–excluded phenotype, and the inflamed phenotype [126]. Each of these profiles seems to be associated with specific pathological mechanisms that may hamper the host immune response’s ability to kill cancer cells [127]. Such profiles can be easily assessed through a comprehensive analysis of spatial immune infiltration patterns (“topography”) and across various immune cell types [128]. A study from our group revealed that this approach is easily applied in routine histological analysis and can detect a subgroup of immune-desert tongue squamous cell carcinoma characterized by a very poor prognosis [129]. However, a simple histologic evaluation has its own limitations and may not be useful for more accurate stratification among different immune cell types.

Recent guidelines of the International Immuno-oncology Working Group have been extensively utilized for the analysis of tumor-infiltrating lymphocytes (TILs) and should be used as a milestone for implementation in clinical practice [130]. More broadly, the identification of specific immune subpopulations of immune cells through the use of molecular biomarkers seems to be a promising approach. An ever-increasing number of studies has been published on this topic, thus raising the need of literature synthesis through data meta-analysis, and good quality evidence is present for some of these immune cell subpopulations. In particular, prognostic studies of OSCC tumors with stromal abundance of CD163+ tumor-associated macrophages (TAMs) evaluated by immunohistochemistry showed a significantly worse prognosis [131].

Another important aspect to take into consideration is the possibility of making a systemic assessment of the tumor immune response by evaluating the population of cells present in the blood. Among these, pre-treatment quantification of the neutrophil-to-lymphocyte ratio significantly impacts the prognosis of head and neck SCC patients [132]. Studies on such a type of prognostic biomarkers are abundant and dispersive, and they represent just an intermediate step toward biomarker implementation in clinical practice [133]. The next step should be to combine multiple predictors in a prognostic model whereby risk of a specific endpoint can be calculated for individual patients [134]. The routine application of such tools requires multiple validations from diverse patient cohorts in different countries and the implementation of their graphical representation for an easier use by physicians [135].

Ultimately, these efforts should be aimed at developing a staging system that includes immune-related features, so that a TNM-Immune Staging System can be implemented [136].

## 5. Conclusions

In this article, we presented evidence supporting the role of immune cells in oral carcinogenesis. Lymphocytes and macrophages are key actors in this process and have gained an important role in OPMDs and in immunoediting. The switch from CD8+ to CD4+ and cytokine profile have been associated with malignant progression. Another mechanism may involve the communication between FoxP3+ CD4+ T lymphocytes, which increase in the progression from OPMD to OSCC, together with the abundanceof CD68+ macrophages in an IL-10-enriched environment. Fibroblasts and secreted molecules including cortisol may exert immunoregulatory functions that are crucial for promoting immune escape and cancer development. So far, the advances in the understanding of the immune function of the TME have led to relatively little improvements in treatment modalities for OSCC and have not been translated into more accurate diagnostic/staging systems. Filling this gap will be instrumental for the development of precision oncology.
